# Annexin A2 Flop-Out Mediates the Non-Vesicular Release of DAMPs/Alarmins from C6 Glioma Cells Induced by Serum-Free Conditions

**DOI:** 10.3390/cells10030567

**Published:** 2021-03-05

**Authors:** Hayato Matsunaga, Sebok Kumar Halder, Hiroshi Ueda

**Affiliations:** 1 Pharmacology and Therapeutic Innovation, Graduate School of Biomedical Sciences, Nagasaki University, Nagasaki 852-8521, Japan; hayatom@nagasaki-u.ac.jp (H.M.); shalder@sdbri.org (S.K.H.); 2 Department of Medical Pharmacology, Graduate School of Biomedical Sciences, Nagasaki University, Nagasaki 852-8523, Japan; 3 San Diego Biomedical Research Institute, San Diego, CA 92121, USA; 4 Department of Molecular Pharmacology, Graduate School of Pharmaceutical Sciences, Kyoto University, Kyoto 606-8501, Japan

**Keywords:** non-vesicular release, SNARE proteins, annexin A2, ATP8A2, DAMPs/alarmins

## Abstract

Prothymosin alpha (ProTα) and S100A13 are released from C6 glioma cells under serum-free conditions via membrane tethering mediated by Ca^2+^-dependent interactions between S100A13 and p40 synaptotagmin-1 (Syt-1), which is further associated with plasma membrane syntaxin-1 (Stx-1). The present study revealed that S100A13 interacted with annexin A2 (ANXA2) and this interaction was enhanced by Ca^2+^ and p40 Syt-1. Amlexanox (Amx) inhibited the association between S100A13 and ANXA2 in C6 glioma cells cultured under serum-free conditions in the in situ proximity ligation assay. In the absence of Amx, however, the serum-free stress results in a flop-out of ANXA2 through the membrane, without the extracellular release. The intracellular delivery of anti-ANXA2 antibody blocked the serum-free stress-induced cellular loss of ProTα, S100A13, and Syt-1. The stress-induced externalization of ANXA2 was inhibited by pretreatment with siRNA for P4-ATPase, ATP8A2, under serum-free conditions, which ablates membrane lipid asymmetry. The stress-induced ProTα release via Stx-1A, ANXA2 and ATP8A2 was also evidenced by the knock-down strategy in the experiments using oxygen glucose deprivation-treated cultured neurons. These findings suggest that starvation stress-induced release of ProTα, S100A13, and p40 Syt-1 from C6 glioma cells is mediated by the ANXA2-flop-out via energy crisis-dependent recovery of membrane lipid asymmetry.

## 1. Introduction

Biological molecules, including damage-associated molecular patterns (DAMPs)/alarmins, are extracellularly released in distinct modes via exocytosis by various mechanisms involving lysosomes (interleukin (IL)-1β, high-mobility group box 1 (HMGB1)), exosomes (galectin-3), exovesicles (galectins and transglutaminase), and transporters (fibroblast growth factor (FGF)-2) [1,2,3,4,5,6,7,8]. The mechanism of stress-induced non-classical release of FGF-1 and IL-1α was initially reported by Prudovsky, Maciaq, and their colleagues [1,9,10], followed by our studies on the release of FGF-1 and prothymosin α (ProTα) [11,12,13,14]. These studies demonstrated that S100A13 plays a key role as a cargo protein by forming multiple protein complexes with FGF-1, IL-1α, or ProTα. The non-classical release is mediated via active machinery since it is completely inhibited by the non-classical inhibitor amlexanox (Amx) with an affinity toward S100A13 [15,16].

p65 synaptotagmin-1 (p65 Syt-1), a Ca^2+^-sensor, plays a role in the classical vesicular release as the vesicular docking partner by binding to syntaxin (Stx)/synaptosome-associated protein of 25 kDa (SNAP-25) that forms a complex with target soluble *N*-ethylmaleimide-sensitive factor-attachment protein receptors (SNAREs). This process is known as exocytosis [17]. In contrast, in the stress-induced non-classical and non-vesicular release of FGF-1 or ProTα, soluble p40 synaptotagmin-1 (p40 Syt-1) lacking the membrane-spanning domain of p65 Syt-1 is released together with S100A13, a cargo protein [15,18]. Our recent study further revealed that the intracellular delivery of anti-Stx-1 IgG or siRNA targeting Stx-1 blocked the serum-free release of Syt-1, S100A13, and ProTα [18]. These findings suggest that p40 Syt-1 may recruit the protein complex of S100A13 and ProTα to the target SNARE at the plasma membranes, analogous to p65 Syt-1 in the vesicular docking machinery in the case of classical exocytosis.

However, as the non-vesicular release employing p40 Syt-1 does not involve the fusion of vesicles to target membranes, the final force driving the extracellular release of these proteins remains to be determined. We speculate that annexin A2 (ANXA2) is another possible target protein for membrane tethering/docking since it is a Ca^2+^-dependent acidic phospholipid (e.g., phosphatidylserine/PS)-binding protein and flopped-out by plasma membrane phospholipid remodeling [19]. In addition, ANXA2 exhibits an affinity for S100A10 [20,21,22], a congener of S100A13, which is a cargo protein for the serum-free -and Ca^2+^-dependent non-classical release of ProTα and FGF-1 [11,18]. Here, we provide evidence for the role of ANXA2 in the non-classical release of the protein complex comprising ProTα, S100A13, and Syt-1.

## 2. Materials and Methods

### 2.1. Materials

Amlexanox (Amx) was kindly supplied by Takeda Pharmaceutical Company Ltd. (Osaka, Japan). ω-Conotoxin GVIA (ω-CTX GVIA) was purchased from Sigma (St. Louis, MO, USA), and ethylene glycol tetraacetic acid (EGTA) was purchased from Nacalai Tesque (Kyoto, Japan).

### 2.2. Antibodies

For immunoblotting, immunoprecipitation, immunocytochemistry or intracellular antibody delivery experiments, the following antibodies were used: mouse anti-prothymosin alpha (ProT*α*) monoclonal antibodies (Clones: 2F11 and 4F4, Alexis Biochemicals, Lausen, Switzerland), rat anti-ProT*α* monoclonal antibody (Clone: 1*–*21) (developed in our laboratory, Nagasaki University, Nagasaki, Japan) [23], N-terminus recognition goat anti-annexin A2 (ANXA2) polyclonal antibody and horseradish peroxidase (HRP)-conjugated mouse anti-β-actin antibody (Santa Cruz Biotechnology Inc., Santa Cruz, CA, USA), rabbit anti-S100A13 antibody (kindly provided by Dr. T. Maciag, Center for Molecular Medicine, Maine Medical Center Research Institute, Scarborough, ME, USA), mouse anti-synaptotagmin-1 (Syt-1) monoclonal antibody (Wako, Osaka, Japan), mouse monoclonal anti-Stx-1 antibody (Wako, Osaka, Japan), rabbit anti-ATP8A2 polyclonal antibody (Abnova, Taipei, Taiwan), and normal mouse or goat IgG (ICN/Cappel Inc., Durham, NC, USA). For in situ proximity ligation assay (PLA) or enzyme-linked immunosorbent assay (ELISA)-based protein binding assay, the following antibodies were used: mouse anti-S100A13 monoclonal antibody (Abnova, Taipei, Taiwan) and rabbit anti-ANXA2 polyclonal antibody (Santa Cruz Biotechnology Inc.).

### 2.3. Gene Constructs and Purification of Recombinant Proteins

The rat ANXA2 gene was amplified from cDNA derived from rat embryonic brain. The following PCR primers were used: ANXA2-Forward, 5′-AGGATCCATGTCTACTGTCCACGAAATC-3′ and ANXA2-Reverse, 5′-AAAGCTTTCAGTCGTCCCCACCACACAG-3′. Forward-primers contain a BamHI site, whereas Reverse-primers contain a stop codon and a HindIII site. His_6_-tagged recombinant protein of ANXA2 was constructed by cloning the amplified genes in-frame into the BamHI–HindIII sites of pQE-30 UA 6xHis-tagging vector (Qiagen, Tokyo, Japan). Recombinant His_6_-ANXA2 protein was purified by using a COSMOGEL^®^ His-Accept agarose (Nacalai Tesque). The plasmid construction of ProTα-enhanced green fluorescent protein (EGFP) for mammalian expression, and the plasmid construction and purification of recombinant *Strep*-tagII-S100A13 protein were performed according to previously described protocol [11,12,14]. The *E. coli* strains DH10B and BL21 (DE3) were transformed with each of these constructs for sub-cloning and protein expression, respectively. His_6_-tagged protein expression was induced by 1 mM isopropyl β-d-1-thiogalactopyranoside at 30 °C for 5 h. All recombinant proteins were dialyzed with interaction buffer (50 mM Tris-HCl pH 7.6, 15 mM NaCl, 140 mM KCl).

### 2.4. C6 Glioma Cell Culture

According to previously described protocol [18], rat C6 glioma cells (American Type Culture Collection^®^, CCL-1017^TM^; Manassas, VA, USA) were cultured in Dulbecco’s Modified Eagle Medium (DMEM) (Nacalai Tesque) supplemented with 10% fetal bovine serum (FBS) (PAA Laboratories GmbH, Pasching, Austria) and 1% penicillin-streptomycin (Sigma). ProTα-EGFP stably-expressing C6 glioma cells were prepared as described earlier [14]. For serum-deprivation stress, the culture medium was replaced by DMEM without FBS.

### 2.5. Primary Culture of Neurons

Primary culture of neurons from the cerebral cortex of 17-day-old embryonic rats was performed according to a previously reported protocol [24,25]. Briefly, cortical tissues were minced into pieces in sterile phosphate-buffered saline (PBS), pH 7.4, and dissociated with 0.25% trypsin (Invitrogen, Carlsbad, CA, USA) and 0.01% DNase I (Sigma-Aldrich, St. Louis, MO, USA) in PBS for 12 min at 37 °C. The reaction was terminated by the addition of 0.25% soybean trypsin inhibitor (Sigma-Aldrich), and the cell suspension was centrifuged at 1000× *g* for 5 min. The pellet was resuspended in serum-supplemented D/F medium (1:1 Dulbecco’s modified Eagle’s medium/Ham’s F-12 medium (Invitrogen) containing 5% horse serum (HS), 5% fetal bovine serum (FBS), and 1% 2-mercaptoethanol (Invitrogen, San Diego, CA, USA)). Dissociated neurons were seeded at a density of 5 × 10^5^ cells/cm^2^ onto poly-DL-ornithine (Sigma-Aldrich)-coated 24-well plates for extracellular ProTα measurement, and subsequently cultured at 37 °C in a 5% CO_2_ atmosphere. Cytosine β-d-arabinofranoside (Ara-C; Sigma-Aldrich) at 0.3 μM was added to the culture at 24 h after seeding, followed by another 48 h of culture.

### 2.6. Oxygen Glucose Deprivation (OGD)–Reperfusion Stress Model

For the OGD stress, cortical neurons cultured in serum-supplemented D/F medium for 7 days (3 days in the case with siRNA treatments) and were washed 3 times with glucose-free balanced salt solution (BSS; 116 mM NaCl, 5.4 mM KCl, 1.8 mM CaCl_2_, 0.8 mM MgSO_4_, and 1 mM NaH_2_PO_4_, pH 7.3), which had been deaerated using a vacuum. After the replacement with BSS, neurons were exposed to hypoxia (<0.4% O_2_, 5% CO_2_, and 95% N_2_) for 2 h at 37 °C in a commercially available culture incubator (Nuair, Tokyo, Japan). After the hypoxia-treatment, the culture medium was returned with fresh D/F medium containing 5% HS and 5% FBS, and the neurons were further incubated for the indicated periods in 5% CO_2_ atmosphere (reperfusion).

### 2.7. Intracellular Antibody Delivery

Two µg of anti-ANXA2 IgG (goat) or goat IgG was added to cultured C6 glioma cells (5 × 10^5^ cells) in a volume of 250 µL of serum-free DMEM containing BioPORTER^®^ protein delivery reagent (Catalog no. BP509604, Gene Therapy Systems Inc., San Diego, CA, USA), according to the manufacturer’s protocol. After incubation for 60 min, the cells were cultured with DMEM containing 10% FBS for 3 h, followed by serum-deprivation for another 3 h and used for immunocytochemistry and measurements of ProTα levels in CM and cells. ProTα-EGFP was efficiently incorporated into C6 glioma cells within 30 min by the use of a BioPORTER^®^ protein delivery system. When fluorescence-labeled protein supplied by the BioPORTER^®^ kit was delivered into C6 glioma cells in a preliminary study, the fluorescence was detected throughout the cell, but not dot-likely distributed.

### 2.8. siRNA Treatments

For the gene knock-down assay, scrambled siRNA as negative control (SIC-001-10, MISSION siRNA Universal Negative Control) [26] or siRNA for ATP8A2 (SASI_Mm02_00323184) were purchased from Sigma. C6 glioma cells were transfected by Lipofectamine™ RNAiMAX reagent (Life Technologies Corp., Tokyo, Japan). Transfected cells were cultured for 72 h at 37 °C prior to use in experiments. siRNAs for syntaxin-1A (Stx-1A) (SASI_Rn01_00065142) and annexin A2 (ANXA2) (SASI_Rn01_00033819) were purchased from Sigma. Primary cultured cortical neurons (4 days in vitro) were transfected by Lipofectamine™ RNAiMAX reagent (Life Technologies Corp., Tokyo, Japan). Transfected cells were cultured for 72 h at 37 °C prior to use in experiments.

### 2.9. Immunocytochemistry

According to previously described protocol [18], cells were fixed in 4% paraformaldehyde (PFA) in phosphate-buffered saline (PBS) for 30 min and then permeabilized with methanol for 10 min at room temperature. After washing with PBS, the fixed cells were incubated with blocking buffer (1% bovine serum albumin (BSA) and 0.1% Triton X-100 in PBS) for 3 h at 4 °C followed by incubation with a primary antibody (1:300 dilution in blocking buffer) overnight at 4 °C. Methanol and Triton X-100 were not used for the ANXA2 externalization assay. The cells were then incubated with a fluorescein isothiocyanate (FITC)- or Cy3- conjugated secondary antibody (1:500 dilution in blocking buffer, Chemicon International Inc., Temecula, CA, USA). The nuclei were visualized with Hoechst 33342 (Molecular Probes, Eugene, OR, USA). Images were captured with a BZ-8000 microscope (Keyence, Osaka, Japan) with a 20× Plan APO lens (Nikon, Tokyo, Japan) or with a LSM 510 META confocal laser microscope with a 40× Plan-Neofluar lens or a 63× Plan-Apochromat lens (Carl Zeiss, Oberkochen, Germany).

### 2.10. Strep-TagII Pull-Down Assay

The pull-down assay was performed as described previously [18]. For the preparation of cytosolic fraction, the cells (4 × 10^5^ cells) were harvested and homogenized with 5 volumes of interaction buffer containing protease inhibitor cocktail (20 μM *p*-(amidinophenyl)methanesulfonyl fluoride (APMSF) and x1 protease inhibitor cocktail; Catalog no. 04080-24; Nacalai Tesque) by a Dounce homogenizer. The nuclear fraction was collected by centrifugation at 1000× *g* for 5 min, and the supernatant was used as the cytosolic fraction. For the *Strep*-tagII pull-down assay, the cytosolic fraction of *Strep*-tagII-S100A13-stably expressing C6 glioma cells was added to 200 μL of StrepTactin™ MicroPrep^®^ resin beads (IBA GmGH, Gottingen, Germany) in the presence or absence of 100 µM CaCl_2,_ and incubated on a rotor for 1 h at 4 °C. After washing three times with interaction buffer with or without 100 µM CaCl_2_, the beads were quenched with 50 µL of SDS sample buffer and 20 μL was used for immunoblotting.

### 2.11. Immunoblotting

For the measurement of ProTα levels in C6 glioma cells treated with and without serum-deprivation and intracellular antibody delivery, the cells were first boiled for 5 min and subjected to 15% SDS-PAGE fractionation and electrotransferred onto nitrocellulose membranes in acidic conditions in 20 mM sodium acetate buffer, pH 5.2, followed by fixation with 0.5% glutaraldehyde. The nitrocellulose membranes were blocked with 5% non-fat milk in TBST (20 mM Tris–HCl, pH 7.6, 150 mM NaCl, 0.1% Tween-20) and probed with the antibody against ProTα (2F11) overnight at 4 °C. The immunoreactive band was visualized using SuperSignal West Pico Chemiluminescent Substrate for detection of horseradish peroxidase (PIERCE, Rockford, IL, USA).

### 2.12. Immunoprecipitation

For immunoprecipitation (i.p.p.t), protein G-Sepharose™ beads (GE Healthcare Bio-Science Corp, Piscataway, NJ, USA)-captured rabbit anti-S100A13 antibody were added to conditioned medium (CM) in the presence of ethylenediaminetetraacetic acid (EDTA) free protease inhibitor cocktail (Nacalai Tesque) and incubated on a rotor for 2 h at 4 °C. The beads were washed three times with medium without serum and quenched with 50 µL of SDS sample buffer.

### 2.13. Kinetic Analysis for Protein-Protein Interaction

The kinetic analysis for protein–protein interaction was performed, as previously reported [18]. We performed an ELISA-based protein binding assay to determine the interaction between *Strep*-tagII-S100A13 and His_6_-ANXA2. *Strep*-tagII-S100A13 was immobilized onto a 96-well Immobilizer™ Streptavidin plate (Nalge Nunc International Corp., Naperville, IL, USA) in the interaction buffer for 1 h at room temperature, and then incubated with blocking buffer (interaction buffer containing 1% BSA). After washing with interaction buffer, the plates were incubated with various concentrations of His_6_-ANXA2 in blocking buffer overnight at 4 °C, and further incubated with a primary antibody against ANXA2 (1:1000 dilution in blocking buffer). The plates were then incubated with HRP-conjugated secondary antibody (1:10,000 in blocking buffer, ZYMED Laboratories, San Francisco, CA, USA). The enhancement of binding potency of His_6_-ANXA2 with *Strep*-tagII-S100A13 by applying His_6_-p40 Syt-1 was also determined by an enzyme-linked immunosorbent assay (ELISA)-based protein binding assay in the presence of 100 µM Ca^2+^. The amount of protein binding to S100A13 was measured using o-phenylenediamine as a substrate, and the absorbance at 492 nm was read with an automatic ELISA analyzer (ImmunoMini NJ-2300, Nalge Nunc International Corp.). Binding unit (BU) represents the value calculated from the observed absorbance at 492 nm (Abs_492_) in the presence of guest protein—Abs_492_ in the absence of guest protein. For kinetic analysis, *K*_D_ was calculated from the Hanes–Woolf Plot: [Conc. of His_6_-ANXA2]/BU = 1/BU_max_ × [Conc. of His_6_-ANXA2] + *K*_D_/BU_max_.

### 2.14. In Situ Proximity Ligation Assay

The detection, visualization and quantification of protein–protein interaction (physical closeness within 40 nm) were performed using Duolink™ in situ proximity ligation assay (in situ PLA) reagents (Olink Bioscience, Uppsala, Sweden), according to the manufacturer’s protocol. In this technology, each protein of the complex in the fixed cell preparation was labeled with an oligonucleotide-conjugated antibody and then used for amplification of the signal by generating a DNA surrogate of the protein using a polymerase. Detailed technology procedures including the principle and method were recently reported elsewhere [27].

### 2.15. Measurement of Intracellular and Extracellular ProTα-EGFP Levels

C6 glioma cells stably expressing ProTα-EGFP were grown on 96-well FluoroNunc™ plates (Nalge Nunc International Corp.). At 24 h after seeding, the medium was changed three times with fresh phenol red-free medium with or without 10% serum, and the cells were then incubated at 37 °C for 3 h. After removal of the supernatant, fresh phenol red-free medium without serum was added and used for the measurement of fluorescent intensity using a 1420 ARVO™ multi label counter (Perkin Elmer Japan Co., Ltd., Yokohama, Japan) with a 14-nm bandwidth excitation filter at 485 nm and a 25-nm bandwidth emission filter at 535 nm. To determine the released ProTα-EGFP, the supernatants were added to 96-well FluoroNunc™ plates and also used for the measurement of fluorescence intensity.

### 2.16. Proximity Extension Assay

The measurement of extracellular native ProTα in conditioned medium was performed by the use of Proseek™ (Olink Bioscience) on the basis of proximity extension assay (PEA) technology [28]. To create Proseek™ probes in PEA, anti-ProTα monoclonal antibodies (clones: 1–21 and 2F11) were conjugated to oligonucleotide probe A and B, respectively. To calculate the extracellular concentration of ProTα, a calibration curve was created by preparing a set of standard solutions with known concentrations of recombinant ProTα protein. Expression and purification of recombinant ProTα was performed according to a previously reported protocol [29].

### 2.17. Statistical Analysis

All statistical analyses were performed using Statcel software (version 4, OMS Publishing Inc., Saitama, Japan) and EZR (version 1.40, Saitama Medical Center, Jichi Medical University, Saitama, Japan). Statcel is an add-in software for performing statistical analysis in Excel (Excel 365, Microsoft, Redmond, WA, USA). EZR is a graphical user interface for R (version 3.6.1., The R foundation for statistical Computing, Vienna, Austria). More precisely, it is a modified version of the R commander designed to add statistical functions frequently used in biostatistics. Data normality was analyzed using the Shapiro–Wilk test. Equality of population variance of multi-groups was analyzed by the Bartlett test. Multiple comparison post-hoc analysis of parametric data was analyzed by the Tukey–Kramer test after testing for equality of mean values by the single-factor analysis of variance (ANOVA). Multiple comparison post-hoc analysis of non-parametric data was tested by the Steel test after analyzing for equality of mean values by the Kruskal–Wallis test. Differences were defined as statistically significant when *p* < 0.05. All results are shown as means ± standard error of the mean (S.E.M.).

## 3. Results

### 3.1. Ca^2+^-Dependent Interaction between ANXA2 and S100A13

**Figure 1:**
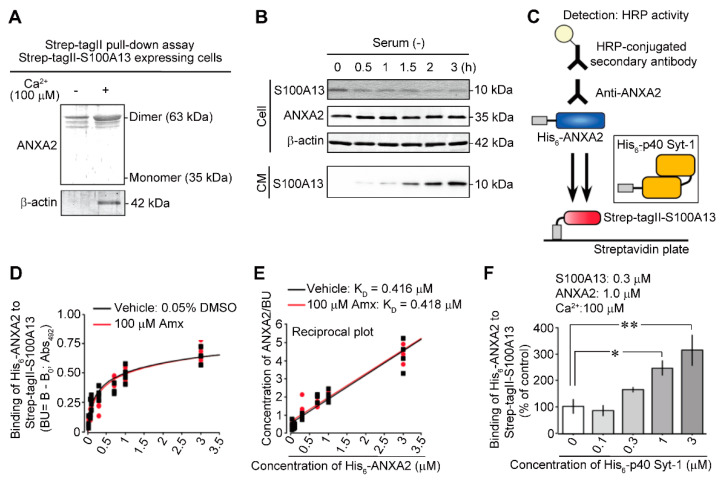
Evidence for Ca^2+^-dependent interaction between annexin A2 (ANXA2) and S100A13 under serum-free conditions. (**A**) Pull-down assay using *Strep*-tagII-S100A13. *Strep*-tagII-S100A13 and C6 glioma cell lysates were incubated in the absence (left lane) and presence (right lane) of 100 μM Ca^2+^. Results represent the immunoblot using anti-ANXA2 IgG (upper lanes) and anti-β-actin antibody (lower lanes). The pull-down assay in Figure 1A showed that ANXA2 dimer interacts with *Strep*-tagII-S100A13 in C6 glioma cell lysates in a Ca^2+^-enhanced manner. In the previously published study [18], we have shown that p40 Syt-1 also interacts with *Strep*-tagII-S100A13 in C6 glioma cell lysates in a Ca^2+^-enhanced manner. As both pull-down assays using *Strep*-tagII-S100A13 have been done with the same C6 glioma cell lysates, it appears that S100A13 forms the same protein complex with both experiments p40 Syt-1 and ANXA2. The experiment was done using same C6 glioma cell lysates as previously reported to show the interaction between *Strep-*tagII-S100A13 and p40 Syt-1 [18]. (**B**) Serum-free-induced release of S100A13, but not ANXA2 from C6 glioma cells. Results represent the time course of protein levels of S100A13, ANXA2 and β-actin in cells (upper panels) and conditioned medium (CM) (lower panel) by immunoblot analysis. Extracellular S100A13 was recovered from CM using immunoprecipitation. (**C**) Schematic model of interaction between His_6_-ANXA2 and *Strep*-tagII-S100A13 bound to Streptavidin plate in an ELISA-based protein binding assay. Inset: enhancement of the interaction in the presence of His_6_-p40 synaptotagmin-1 (Syt-1). (**D**,**E**) No effect of amlexanox (Amx) on the His_6_-ANXA2 binding to *Strep*-tagII-S100A13 in terms of analyses of His_6_-ANXA2 concentration-dependency (**D**) and its reciprocal plot (**E**). (**F**) His_6_-p40 Syt-1 concentration-dependent enhancement of His_6_-ANXA2 binding to *Strep*-tagII-S100A13 in the presence of Ca^2+^. Data are presented as the mean ± standard error of the mean (S.E.M.) from a Tukey–Kramer multiple comparison test. * *p* < 0.05 and ** *p* < 0.01. *n* = 4 experiments per group.

To examine whether ANXA2 associates with S100A13, the cargo protein mediating stress-induced non-classical release under serum-free conditions [11,14], we first performed a pull-down assay using the lysates of C6 glioma cells expressing *Strep*-tagII-S100A13 in the absence or presence of 100 μM of Ca^2+^. As shown in Figure 1A, dimeric ANXA2 of 63 kDa interacted with *Strep*-tagII-S100A13, which was enhanced by Ca^2+^. Several smaller immunoreactive bands may represent partially cleaved ANXA2 proteins; however, the identification remains unclear. The pull-down efficiency seems to be equivalent between the samples in the absence or presence of Ca^2+^. This finding can be attributed to the fact that a significant difference was not observed in the efficiency of pull-down in the absence and presence of Ca^2+^ in terms of the amount of input Strep-tag II-S100A13 and native S100A13. S100A13 was demonstrated as a dimer partner in a previous study using the same C6 glioma lysates both in the absence and presence of Ca^2+^ [18], which were used also in the present study. Similarly, β-actin interacted with *Strep*-tagII-S100A13 in a Ca^2+^-enhanced manner. Annexins reversibly bind to the actin cytoskeleton and are involved in exocytosis and endocytosis [30,31]. The Ca^2+^-dependent pull-down of β-actin is likely due to the association of ANXA2 with *Strep*-tagII-S100A13. This finding is consistent with reports stating that the S100 gene family is associated with the cytoskeleton and is responsible for transmembrane signaling and secretion [32,33,34]. When the intracellular and extracellular cell contents of S100A13 were examined using immunoblotting analysis at different time points after replacement with the serum-free medium, the release of S100A13 was initiated after 0.5 h, as shown in Figure 1B. However, there was no change in the levels of ANXA2 or β-actin. ANXA2 present in the cell extracts was detected as a single band (Figure 1B). However, the protein bound to S100A13 was detected only in the dimer form (Figure 1A), which is consistent with the results of a previous report [18]. As the amount of ANXA2 dimers bound to S100A13 seemed to be very low compared to the cell contents, significantly smaller amounts of partially cleaved dimer ANXA2 bands were also detectable (Figure 1A). It should be also noted that the detection of 63 kDa bands in the sodium dodecyl sulfate-polyacrylamide gel electrophoresis (SDS-PAGE) reflects that ANXA2 dimer binding was very strong; hence, each protein was not separated by SDS-PAGE.

As shown in Figure 1C, the interaction of His_6_-ANXA2 with *Strep*-tagII-S100A13 immobilized onto the streptavidin plate was evaluated using an ELISA assay. Figure 1D,E show the binding profile of His_6_-ANXA2 with *Strep*-tagII-S100A13 immobilized on the plate and its reciprocal plot. The addition of amlexanox (Amx) at 100 µM did not affect the binding of either protein; however, Amx abolished the stress-induced release of S100A13, FGF-1, and ProTα under serum-free conditions [11,14]. In contrast, the binding of His_6_-ANXA2 to immobilized *Strep*-tagII-S100A13 increased in a concentration-dependent manner following the addition of p40 Syt-1 in the presence of 100 µM of Ca^2+^ (Figure 1F). The p40 Syt-1 enhanced the interaction between His_6_-ANXA2 and Strep-tagII-S100A13. This process may be dependent on Ca^2+^ since the interaction between S100A13 and p40 Syt-1 is also dependent on Ca^2+^ [18]. Small but significant amounts of ANXA2 dimers were bound to Strep-tag II-S100A13 (Figure 1A, left lane), and significant binding of His_6_-ANXA2 to Strep-tagII-S100A13 (Figure 1D) was detected in the absence of Ca^2+^. Hence, the enhancement may be attributed to a mechanism that stabilizes the structure of S100A13 via binding to p40 Syt-1 in the presence of Ca^2+^. Therefore, the interaction with ANXA2 is strengthened.

### 3.2. Involvement of Stress-Induced ANXA2 Flop-Out in the Co-Release of S100A13 and ProTα

**Figure 2:**
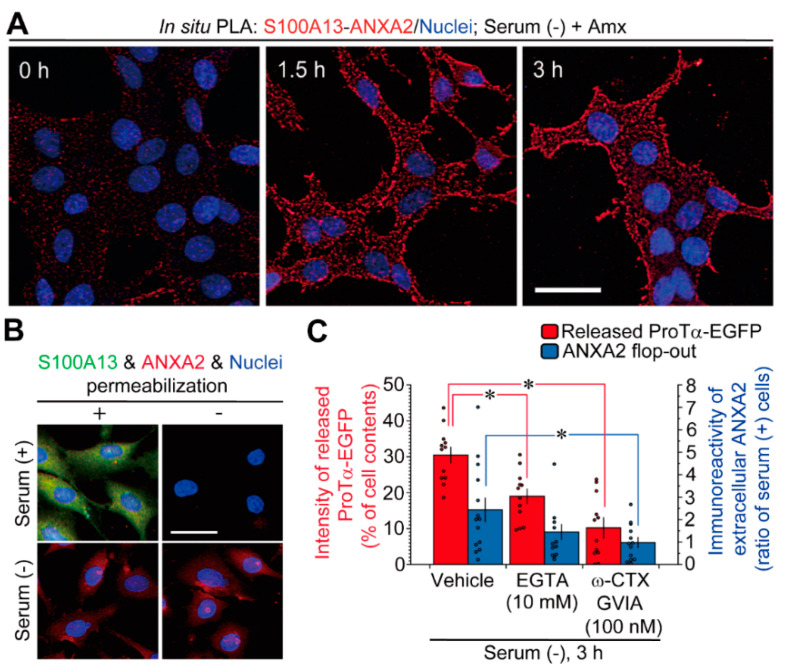
Roles of annexin A2 flop-out in non-classical release of S100A13 and ProTα under serum-free conditions. (**A**) In situ proximity ligation assay (PLA) shows the evidence for stress-induced interaction between S100A13 and annexin A2 (ANXA2) in the presence of amlexanox (Amx). (**B**) ANXA2 flop-out on the cell surface and S100A13 extracellular release under serum-free conditions. Immunocytochemistry was performed using C6 glioma cells with and without permeabilization. Immunocytochemical analyses with and without permeabilization show the presence of immunoreactive (ir)ANXA2, but not irS100A13 in C6 glioma cells following serum-free stress. (**C**) Involvement of the N-type Ca^2+^ channel in prothymosin alpha (ProTα) release and ANXA2 flop-out under serum-free conditions. Blockade of stress-induced ProTα-EGFP release and increased irANXA2 externalization by the treatment of ethylene glycol tetraacetic acid (EGTA) or ω-conotoxin (ω-CTX) GVIA. Data represent the means ± S.E.M. from a Tukey–Kramer multiple comparison test. * *p* < 0.05 versus the vehicle. For ProTα-EGFP: *n* = 12 experiments per group. For ANXA2 flop-out: *n* = 14 experiments (Vehicle), *n* = 14 experiments (EGTA), *n* = 13 experiments (ω-CTX GVIA). Scale bars, 20 µm.

To evaluate the association of S100A13 and ANXA2 induced by serum-free conditions in C6 glioma cells, we adopted the in situ PLA technique, which could amplify the fluorescent signal owing to the interaction between S100A13 and ANXA2. Furthermore, 100 µM of Amx was added to the culture to block the stress-induced release of S100A13. As shown in Figure 2A, the in situ PLA signals were markedly observed at 1.5 and 3 h after replacement with serum-free medium. These signals were more evident at the edge of cells; however, strong signals were not detected before the application of stress.

Fluorescence microscopic analysis detected both immunoreactive (ir)S100A13 and irANXA2 in permeabilized C6 glioma cells in the presence of serum, as shown in Figure 2B. irANXA2 and irS100A13 were not detected in the absence of permeabilization, indicating that both proteins were present inside the cell. When cells were cultured under serum-free conditions, irS100A13 was not detected in either case with or without permeabilization, indicating that S100A13 is released extracellularly. In contrast, irANXA2 was detected in both cases, suggesting that ANXA2 is externalized under serum-free conditions but remains on the outer surface of C6 glioma cells.

As shown in Figure 2C, the release of prothymosin alpha (ProTα)-EGFP from C6 glioma cells was significantly inhibited by treatment with ethylene glycol tetraacetic acid (EGTA). Treatment with EGTA partially, but not significantly, inhibited the externalization of ANXA2 (Figure 2C). Both ProTα-EGFP release and ANXA2 externalization in C6 glioma cells were significantly inhibited by the addition of ω-conotoxin (ω-CTX) GVIA, an N-type voltage-dependent Ca^2+^ channel inhibitor (Figure 2C).

### 3.3. Blockade of the Release of Syt-1, S100A13, and ProTα via Intracellular Delivery of Anti-ANXA2 Antibody

**Figure 3:**
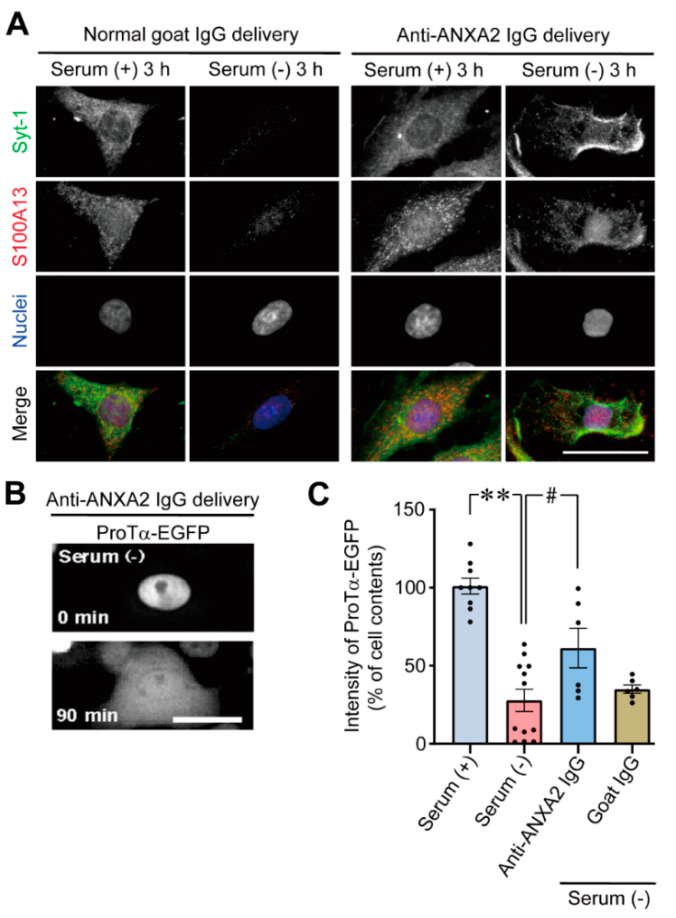
Involvement of annexin A2 inSyt-1, S100A13 and ProTα release under serum-free conditions. (**A**) Blockade of stress-induced release of Syt-1 and S100A13 from C6 glioma cells by intracellular delivery of anti-annexin A2 (ANXA2) IgG. Results represent the representative images of immunoreactive Syt-1 and S100A13 localization by intracellular delivery of normal goat IgG (left panels) and anti-ANXA2 IgG delivery (right panels) under the conditions of serum (+) or serum (−). (**B**) Blockade of stress-induced release of ProTα-EGFP from C6 glioma cells by intracellular delivery of anti-ANXA2 IgG. Results represent the representative images of ProTα-EGFP by anti-ANXA2 IgG delivery. (**C**) Reversal of stress-induced loss of ProTα cell contents by intracellular delivery of anti-ANXA2 IgG (goat), but not by the goat IgG delivery in C6 glioma cells. Results represent the cellular levels of ProTα-EGFP intensity by indicated treatments, in % levels compared with serum (+). Data are presented as the mean ± S.E.M. from a Tukey–Kramer multiple comparison test. ** *p* < 0.01 and # *p* < 0.05. *n* = 9 experiments (serum-containing conditions), *n* = 12 experiments (serum-free conditions), *n* = 6 experiments (goat anti-ANAX2 IgG under serum-free conditions), *n* = 6 experiments (goat IgG under serum-free conditions). Scale bars, 20 µm.

The functional roles of ANXA2 were examined via the intracellular delivery of anti-ANXA2 antibody (Figure 3A). In this experiment both intracellular signals of synaptotagmin-1 (Syt-1) and S100A13 were reduced in C6 glioma cells cultured under serum-free conditions following normal goat antibody delivery. In contrast, both Syt-1 and S100A13 remained in the C6 glioma cells cultured under serum-free conditions following anti-ANXA2 antibody delivery. However, they were localized to the edge of the cells, suggesting that the translocation of the protein complex to the plasma membrane induced under serum-free conditions was not affected by the delivery of anti-ANXA2 antibody. Similarly, the intracellular delivery of anti-ANXA2 antibody inhibited the cellular loss of ProTα-EGFP, resulting in the redistribution of ProTα throughout C6 glioma cells (Figure 3B). However, the localization of ProTα-EGFP to the edge of the cell was not observed, possibly because recombinant ProTα-EGFP is overexpressed in C6 glioma cells. In our previous study [18], we observed negative effects of intracellular IgG delivery on the stress-induced loss of S100A13 and ProTα under serum-free conditions. The stress-induced release of ProTα under serum-free conditions was also evaluated by the stress-induced loss of cellular content. As shown in Figure 3C, the cellular content of ProTα was reduced to 27.5% under serum-free conditions. The loss of ProTα was significantly reversed by the intracellular anti-ANXA2 IgG (goat) delivery (60.6%), but not by goat IgG delivery (34.6%) (Figure 3C). This finding was also supported by the results of a study that showed that ANXA2 siRNA treatment abolished the stress-induced extracellular release of ProTα under serum-free conditions, which was analyzed by proximity extension assay using Proseek™ (Olink Bioscience) technology [18], as shown in Supplementary Figure S1.

### 3.4. Involvement of ATP8A2 in Stress-Induced Flop-Out of ANXA2 under Serum Free Conditions

**Figure 4:**
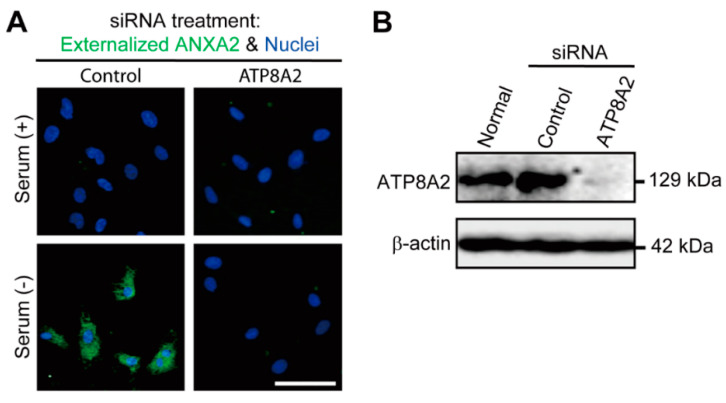
ATP8A2 is involved in flop-out of annexin A2 under serum-free conditions. (**A**) Loss of annexin A2 (ANXA2) flop-out by the treatment of siRNA for ATP8A2. C6 glioma cells without permeabilization were treated with siRNA for ATP8A2, and ANXA2 immunocytochemistry was performed at 3 h after serum-free stress. Images are representative of at least 3 independent experiments. (**B**) Immunoblot evidence for the knock-down of ATP8A2 by siRNA treatment in C6 glioma cells. Scale bars, 20 µm.

To determine whether P4-ATPases are involved in stress-induced externalization of ANXA2, C6 glioma cells were treated with different siRNAs specific for P4-ATPases. As shown in Figure 4A, immunocytochemistry for ANXA2 was performed without permeabilization in siRNA-treated C6 glioma cells. The irANXA2 was not detected in any of the siRNA-treated C6 glioma cells in the presence of serum (3 h). In contrast, under serum-free conditions, ANXA2 externalization was observed at 3 h in control siRNA-treated C6 glioma cells. Treatment with siRNA corresponding to ATP8A2 completely inhibited the ANXA2 externalization 3 h after culturing in the serum-free medium. Treatment with siRNA specific for other P4-ATPases, such as ATP8A1, ATP8B1, ATP8B2, ATP8B3, ATP8B4, and ATP10A, failed to show any effect on the stress-induced externalization of ANXA2 under serum free conditions (Supplementary Figure S2). Immunoblot analysis confirmed the effectiveness of ATP8A2 siRNA treatment on the protein levels of ATP8A2 (129 kDa), but not on those of β-actin (42 kDa) (Figure 4B).

### 3.5. Knock-Down of Key Molecules Prevents Ischemia-Reperfusion Stress-Induced Release of ProTα from Neurons

**Figure 5:**
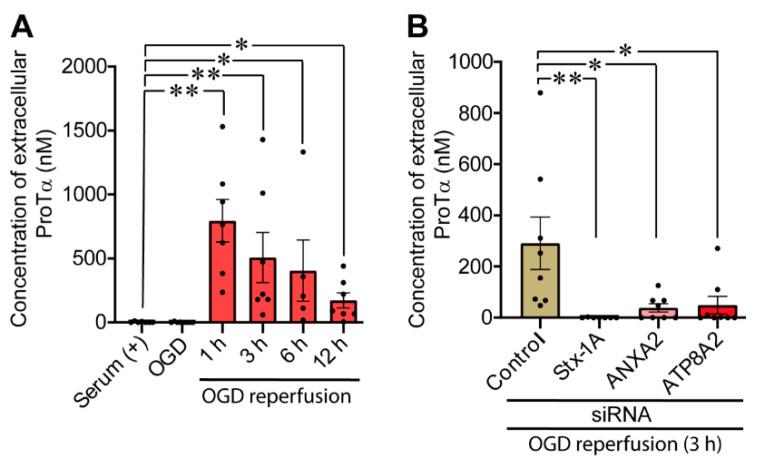
Knock-down of key molecules prevents ischemia-induced release of ProTα from neurons. (**A**) Time course of extracellular release of ProTα from cortical neurons in primary culture after the oxygen glucose deprivation (OGD)–reperfusion stress. Neurons were seeded on 24-well plate at a density of 5 × 10^5^ cells/cm^2^ and the volume of conditioned medium for normal and OGD treatment was 500 μL. Conditioned medium (CM) was collected 2 h after OGD treatment and at various time points after reperfusion and the concentration of ProTα in the medium was measured by proximity extension assay, as previously described [18]. Results show the concentration of ProTα released from 9.5 × 10^5^ neurons in 500 μL of conditioned medium. To calculate the extracellular concentration of ProTα; a calibration curve was created by preparing a set of standard solutions with known concentrations of recombinant ProTα protein. ProTα was rapidly released after reperfusion. Data are presented as the mean ± S.E.M. from a Steel test. * *p* < 0.05 and ** *p* < 0.01. *n* = 7 experiments (serum (+)), *n* = 8 experiments (2 h after OGD), *n* = 7 experiments (1 h after reperfusion), *n* = 7 experiments (3 h after reperfusion), *n* = 5 experiments (6 h after reperfusion), *n* = 7 experiments (12 h after experiments). (**B**) Evidence for the blockade of OGD-reperfusion stress-induced extracellular release of ProTα by the gene knock-down of Stx-1A, ANXA2 and ATP8A2. CM was collected from siRNA-transfected cortical neurons 3 h after reperfusion and the concentration of ProTα was measured. Data are presented as the mean ± S.E.M. Steel test. * *p* < 0.05 and ** *p* < 0.01. *n* = 8 experiments (Control), *n* = 7 experiments (Stx-1A), *n* = 8 experiments (ANXA2), *n* = 8 experiments (ATP8A2).

Neurons are the largest source of extracellularly released ProTα in the brain during ischemic stress [35]. When rat cortical neurons in primary culture were subjected to oxygen glucose deprivation (OGD)-ischemia and reperfusion stress, there was a ProTα release from neurons, measuring by proximity extension assay though 12 h and at a peak time point of 1 h after the reperfusion (Figure 5A). When the cultured neurons were pretreated with siRNAs for Stx-1A, ANXA2 and ATP8A2, ischemia-reperfusion stress-induced ProTα release was largely abolished (Figure 5B).

## 4. Discussion

Before the present study, we described the beneficial effects of prothymosin alpha (ProTα) as a DAMP/alarmin [36,37,38,39,40,41,42]. The starvation stress-induced release of ProTα was mediated before the destruction of the cell membrane [43]. Cellular and molecular studies, including those involving pull-down assays performed with cell lysates or conditioned medium, revealed that S100A13 is involved in Ca^2+^-dependent interaction and co-release [11,12,13,14]. Based on previous studies performed by other groups [1,15,44,45] and our studies [11,12,13,14], S100A13 could be a cargo protein of non-classical and non-vesicular release of FGF-1 as well as ProTα. Further studies also revealed that S100A13 interacts with p40 synaptotagmin-1 (Syt-1) devoid of the membrane-spanning domain of p65 Syt-1, and the S100A13-p40 Syt-1 complex is associated with the target soluble ***N***-ethylmaleimide-sensitive factor-attachment protein receptor (SNARE) protein syntaxin-1 (Stx-1) in a Ca^2+^-dependent manner [13,18]. The finding that amlexanox (Amx) blocks the stress-induced extracellular release of these protein complexes without any influence of the binding between S100A13 and p40 Syt-1 is crucial [13,14,15,18,46]. This indicates that the non-classical release of protein complexes comprising ProTα, S100A13, and p40 Syt-1 occurs actively, owing to the loss of membrane asymmetry. Thus, as the mechanism underlying the S100A13-mediated non-classical and non-vesicular release has been partly elucidated, the purpose of the present study was to investigate the final step, the force driving the transport of protein complexes through the cell membrane.

The clue to solving this question was obtained by reports demonstrating the affinity of S100A10, a congener of S100A13, for ANXA2 [47,48,49]. This interaction, in the presence of Ca^2+^, has been implicated in the organization of membrane domains, lipid rafts, and membrane–cytoskeleton contacts [50,51]. The present study provided evidence that Strep-tagII-S100A13 binds to β-actin as well as the ANXA2 dimer, which is enhanced by Ca^2+^. Considering that S100A13 forms a complex with p40 Syt-1 that further interacts with Stx-1 in a Ca^2+^-dependent manner [18], the present evidence demonstrating the Ca^2+^-enhanced interaction of S100A13 with ANXA2 and β-actin suggests that S100A13 hetero-oligomer translocates to the plasma membrane on the β-actin-rail via ANXA2/acidic phospholipid interaction and p40 Syt-1/Stx-1 interaction. This hypothesis is strengthened by the finding that the interaction between Strep-tagII-S100A13 and His_6_-ANXA2 was enhanced by the addition of His6-p40 Syt-1. These findings are consistent with studies showing that annexins, including ANXA2, reversibly bind to various cell membrane components and the actin cytoskeleton and are involved in cellular processes related to exocytosis and endocytosis [30,31].

Immunoblot analysis result showing that S100A13, but not ANXA2, is lost in C6 glioma cells under serum-free conditions is noteworthy. This finding was further supported by an in situ PLA study and immunocytochemistry analysis with or without permeabilization (Figure 2A,B). In the presence of Amx, which blocks the release of S100A13-containing hetero-oligomers, the stress induced under serum-free conditions enhanced the interaction between S100A13 and ANXA2, as shown in the in situ PLA study. Interestingly, the interaction between S100A13 and ANXA2 was observed in a dotted, but not diffused manner (Figure 2A). However, the identification of the structure to which the protein complex is attached should be examined in future studies. Immunocytochemical analysis showed that irANXA2 was detected in unpermeabilized C6 glioma cells exposed to serum-free conditions, as well as in permeabilized cells under normal conditions. This observation suggests that ANXA2 contributes to the release of S100A13-containing hetero-oligomers. However, this protein itself remains on the outer surface of the cell. The role of ANXA2 in the non-classical release of hetero-oligomers containing S100A13, ProTα, and Syt-1 was demonstrated in a study performed via the intracellular delivery of anti-ANXA2 antibody into C6 glioma cells, as shown in Figure 3. As ethylene glycol tetraacetic acid (EGTA) and w-conotoxin (ω-CTX) blocked the ProTα release and ANXA2 externalization, all these activities may be performed by the stress-induced Ca^2+^ influx under serum-free conditions (Figure 2C), as previously reported [18].

**Figure 6:**
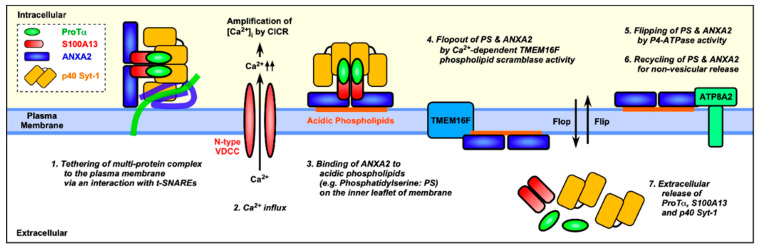
Schematic illustration for the mechanism of stress-induced non-vesicular release of ProTα and S100A13. A multi-protein complex consisting of ProTα (green), S100A13 (red), p40 Syt-1 (yellow) and ANXA2 (blue) is Ca^2+^-dependently assembled. This complex is tethered to the plasma membrane via an interaction between p40 Syt-1 and t-soluble N-ethylmaleimide-sensitive factor-attachment protein receptors (SNAREs), and then binds to the cytoplasmic leaflet of phospholipids via the Ca^2+^-dependent interaction between ANXA2 and phosphatidylserine (PS). Transmembrane protein 16F (TMEM16F), a phospholipid scramblase, causes an externalization of PS and flop-out of ANXA2 in a Ca^2+^-dependent manner. Recruitment of ANXA2 results in the extracellular release of ProTα, S100A13 and p40 Syt-1 in a non-vesicular route. P4-ATPase (ATP8A2 in C6 glioma cells) contributes to recycling of molecules in the non-vesicular release mechanism by flipping of PS and ANXA2.

The asymmetrical localization of phosphatidylserine (PS) is maintained by P4-type ATPases (flippases). Once the cell is exposed to the stress induced under serum-free conditions, which leads to an energy crisis in the cell, many PS molecules move back to the outer leaflet for even distribution in the lipid bilayer. The stress induced under serum-free conditions leads to Ca^2+^influx and mediates the Ca^2+^-dependent formation of protein complexes comprising S100A13, ProTα, and p40-Syt-1 and enhances its association with the plasma membrane via an interaction between p40 Syt-1 and target SNAREs [18]. As ANXA2 has an affinity to acidic phospholipid PS and is associated with S100A13 in a Ca^2+^-dependent manner [52,53], it is speculated that the stress induced under serum-free conditions may lead to the externalization of ANXA2-bound S100A13, ProTα, and p40-Syt-1 complex via the movement of PS. Thus, at first glance, it seems that the knockdown of P4-type ATPase itself may lead to the ANXA2 externalization. However, the present result revealed that the treatment with ATP8A2 siRNA itself did not lead to the externalization. On the contrary, it blocked the stress-induced ANXA2 externalization under serum-free conditions. These results suggest that ATP8A2 knockdown may reverse the asymmetrical localization of PS, but does not cause ANXA2 externalization, since Ca^2+^influx required for ANXA2-containing protein complex formation may not occur in the absence of the serum-free conditions. Rather, it may be presumed that the preceding loss of asymmetrical localization of PS by ATP8A2 knockdown abolished the driving force of serum free-conditions that induced PS movement toward the outer leaflet (Figure 6). In the present study, we successfully obtained evidence of the involvement of ATP8A2, but not other P4-type ATPases, through experiments employing siRNA-mediated knockdown (Supplementary Figure S2). The involvement of ATP8A2 and other key molecules in the stress-induced non-classical release machineries was further evidenced by the experiment, in which knockdown with siRNAs specific for ATP8A2 as well as Stx-1A and ANXA2 abolished the oxygen-glucose deprivation (OGD)-induced ProTα release from rat cortical neurons (Figure 5). However, a previous report states that Ca^2+^-dependent transmembrane protein 16F (TMEM16F) phospholipid scramblase [54], another candidate responsible for the asymmetrical localization of PS, is required for the externalization of ANXA2 and ANXA5 [19]. Hence, further detailed studies on the involvement of other P4-type ATPases and TMEM16F are required for observing serum-free stress-induced ANXA2 externalization and ProTα release under serum-free conditions.

In conclusion, we found that S100A13, a cargo protein involved in the non-classical release of ProTα and p40 Syt-1, interacts with ANXA2 with an affinity for acidic phospholipids. The present study proposes a new mechanism of ANXA2-mediated flopping of the protein complex containing S100A13 through the cell membrane under serum-free conditions or via starvation stress. The initial step involves the energy crisis-induced impairment of the activity of P4-ATPase flippase, ATP8A2, which maintains the inner leaflet-specific asymmetrical distribution of acidic phospholipids. Thus, the non-classical release of the protein complex occurs along with the flopping of acidic phospholipid following the energy crisis.

## Data Availability

The data presented in this study are available in this article.

## Supplementary Materials

The following are available online at https://www.mdpi.com/2073-4409/10/3/567/s1, Figure S1: The extracellular release of ProTα and S100A13 under serum-free stress was abolished by *ANXA2* knock-down, Figure S2: Lack of effects by the treatments of siRNA specific for ATP8A1, ATP8B1, ATP8B2, ATP8B3, ATP8B4 or ATP10A on the serum-free stress-induced externalization of ANXA2 in C6 glioma cells, Supplementary References.

## Abbreviations

Amx: amlexanox; ANXA2, annexin A2; ω-CTX, ω-conotoxin; DAMPs, damage-associated molecular pattern molecules; EGTA, ethylene glycol tetraacetic acid; IR, immunoreactive; in situ PLA, in situ proximity ligation assay; ProTα, prothymosin alpha; SNARE, soluble *N*-ethylmaleimide-sensitive factor attachment protein receptor; Syt-1, synaptotagmin-1.
