# Supplementary material for: Annexin A2 Flop-Out Mediates the Non-Vesicular Release of DAMPs/Alarmins from C6 Glioma Cells Induced by Serum-Free Conditions
Source: Cells. 2021 Mar 5;10(3):567. doi: 10.3390/cells10030567 (PMC7998613; doi:10.3390/cells10030567)
Supplement: Supplementary file 1 [file cells-10-00567-s001.pdf]

Supplemental Information

Annexin A2 flop-out mediates the serum free-induced non-vesicular release of DAMPs/alarmins from C6 glioma cells

Hayato Matsunaga[1, 2†], Sebok Kumar Halder[1, 3†] and Hiroshi Ueda[1,4*]

1. Pharmacology and Therapeutic Innovation, Graduate School of Biomedical Sciences, Nagasaki University, Nagasaki 852-8521, Japan; hayatom@nagasaki-u.ac.jp (H.M.); shalder@sdbri.org (S.K.H.); ueda1qoocai@gmail.com (H.U.)
2. Department of Medical Pharmacology, Nagasaki University of Graduate School of Biomedical Sciences, Nagasaki 852-8523, Japan; hayatom@nagasaki-u.ac.jp (H.M.)
3. San Diego Biomedical Research Institute, San Diego, CA 92121, USA; shalder@sdbri.org (S.K.H.)
4. Department of Molecular Pharmacology, Kyoto University Graduate School of Pharmaceutical Sciences, Kyoto 606-8501, Japan; ueda1qoocai@gmail.com (H.U.)

* Correspondence: E-mail: ueda1qoocai@gmail.com; Tel: +81-75-753-4536

† These authors contributed equally to this work.

Supplementary Figures

Supplementary Figure 1

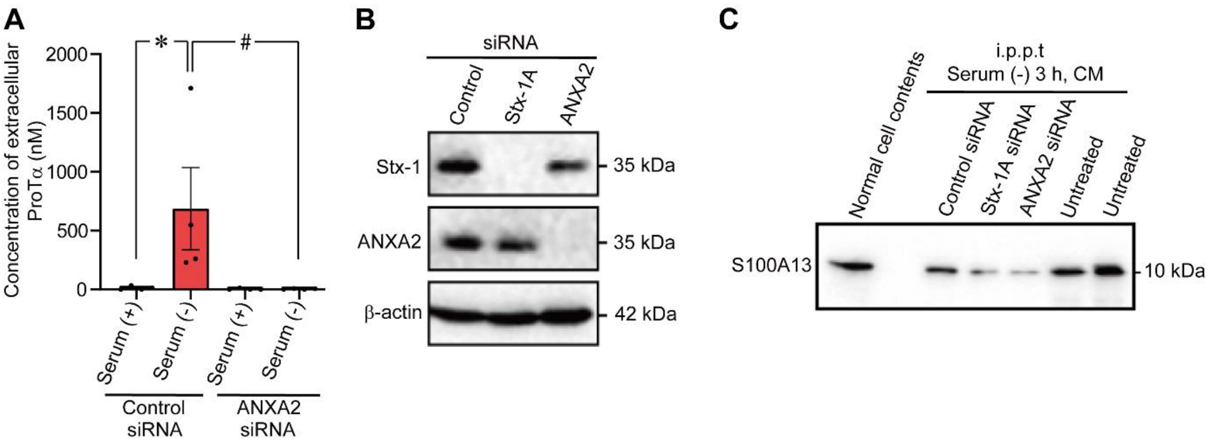

Supplementary Figure 1. The extracellular release of ProTα and S100A13 under serum-free stress was abolished by *ANXA2* knock-down. (A) The extracellular release of ProTα. Conditioned medium of C6 glioma cells was collected after 3 h of serum-free stress and the concentration of ProTα was measured by proximity extension assay. Data are presented as the mean ± S.E.M. Tukey–Kramer multiple comparison test. *$p < 0.05$ and #$p < 0.05$. n = 4 experiments per group. (B) Using immunoblot analysis, the suppression of ANXA2 protein expression was confirmed by siRNA treatment for ANXA2 in C6 glioma cells. Immunoblot analysis with Stx-1A siRNA has been previously reported [1]. (C) The extracellular release of S100A13. Immunoblot shows the extracellular release of S100A13 under serum-free conditions and inhibition of its release by the treatments of siRNA treatments for Stx-1A and ANXA2 in C6 glioma cells. Extracellular S100A13 was recovered from conditioned medium (CM) using immunoprecipitation.

Supplementary Figure 2

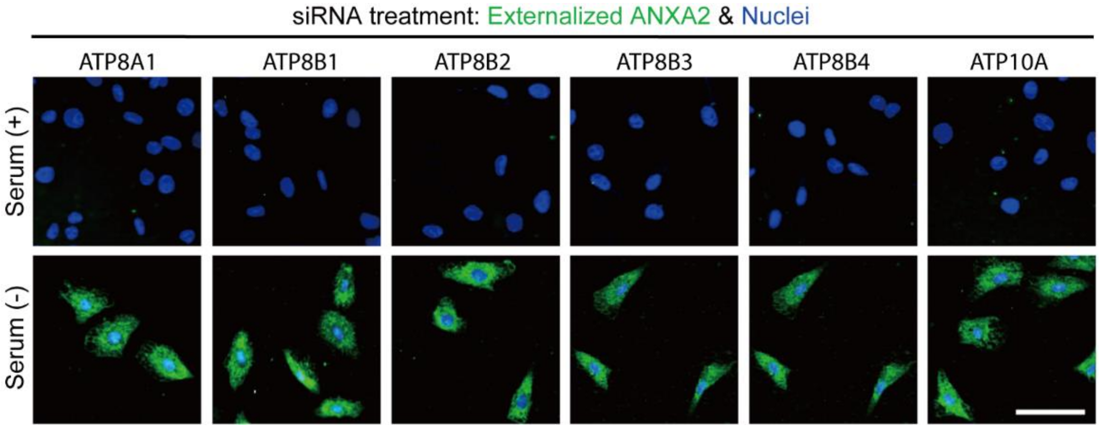

Supplementary Figure 2. Lack of effects by the treatments of siRNA specific for ATP8A1, ATP8B1, ATP8B2, ATP8B3, ATP8B4 or ATP10A on the serum-free stress-induced externalization of ANXA2 in C6 glioma cells. C6 glioma cells without permeabilization were treated with siRNA for ATP8A1, ATP8B1, ATP8B2, ATP8B3, ATP8B4 or ATP10A. ANXA2 immunocytochemistry was performed at 3 h after the serum-free stress. Results show the representative pictures from at least 3 independent experiments. Scale bars, 20 μm.

Supplementary References

1. Matsunaga, H.; Halder, S.K.; Ueda, H. Involvement of SNARE Protein Interaction for Non-classical Release of DAMPs/Alarmins Proteins, Prothymosin Alpha and S100A13. *Cell Mol Neurobiol* **2020**, 10.1007/s10571-020-00950-y, doi:10.1007/s10571-020-00950-y.
